# Alteration of network connectivity in stroke patients with apraxia of speech after tDCS: A randomized controlled study

**DOI:** 10.3389/fneur.2022.969786

**Published:** 2022-09-15

**Authors:** Jiayi Zhao, Yuanyuan Li, Xu Zhang, Ying Yuan, Yinan Cheng, Jun Hou, Guoping Duan, Baohu Liu, Jie Wang, Dongyu Wu

**Affiliations:** ^1^Department of Rehabilitation, Wangjing Hospital, China Academy of Chinese Medical Science, Beijing, China; ^2^Department of Rehabilitation, Xuanwu Hospital Capital Medical University, Beijing, China

**Keywords:** apraxia of speech (AOS), electroencephalogram–EEG, functional connectivity, network, primary motor cortex (M1), stroke, transcranial direct current stimulation (tDCS)

## Abstract

**Objective:**

This study aimed to examine the changes in the functional connectivity of the cortical speech articulation network after anodal transcranial direct current stimulation (A-tDCS) over the left lip region of the primary motor cortex (M1) in subacute post-stroke patients with apraxia of speech (AoS), and the effect of A-tDCS on AoS.

**Methods:**

A total of 24 patients with post-stroke AoS were randomized into two groups and received A-tDCS over the left lip region of M1 (tDCS group)/ sham tDCS (control group) as well as speech and language therapy two times per day for 5 days. Before and after the treatment, the AoS assessments and electroencephalogram (EEG) were evaluated. The cortical interconnections were measured using the EEG non-linear index of cross approximate entropy (C-ApEn).

**Results:**

The analysis of EEG showed that, after the treatment, the activated connectivity was all in the left hemisphere, and not only regions in the speech articulation network but also in the dorsal lateral prefrontal cortex (DLPFC) in the domain-general network were activated in the tDCS group. In contrast, the connectivity was confined to the right hemisphere and between bilateral DLPFC and bilateral inferior frontal gyrus (IFG) in the control group. In AoS assessments, the tDCS group improved significantly more than the control group in four of the five subtests. The results of multivariate linear regression analyses showed that only the group was significantly associated with the improvement of word repetition (*P* = 0.002).

**Conclusion:**

A-tDCS over the left lip region of M1 coupled with speech therapy could upregulate the connectivity of both speech-specific and domain-general networks in the left hemisphere. The improved articulation performance in patients with post-stroke AoS might be related to the enhanced connectivity of networks in the left hemisphere induced by tDCS.

**Clinical trial registration:**

ChiCTR-TRC-14005072.

## Introduction

Apraxia of speech (AoS) is a motor speech disorder, which is characterized by impairment at the motor programming level. Motor programming has long been recognized as a critical step in the speech production process, allowing for the transformation of abstract linguistic codes into specific movement commands interpretable by the motor system (1). Patients with AoS had articulatory difficulties in their speech and prosodic disruptions, identified as an overall slow rate of speech, segmentation of syllables, distorted sounds, consistent error type, abnormal prosody, and increased difficulty with increased length and complexity of utterances (2). Speech and language therapy (SLT) at high intensity, high dose, or for a long time may be beneficial for AoS. Given the high dropout rate shown in a systematic review (3), the recovery of patients with AoS was unsatisfactory.

Transcranial direct current stimulation (tDCS), as a new advance in the treatment, has been proven beneficial for the recovery of AoS (4–6). Recent studies have most consistently suggested the crucial role of the left premotor cortex (PM) and primary motor cortex (M1) for AoS (7–11). In both neurodegenerative and post-stroke AoS, MRI analysis revealed the most common lesion areas spanning the left PM and M1 (8). A study of Duffau H using intraoperative functional mapping in awake patients found that the electrical stimulation of left PM induced transient speech disturbances of counting and naming which were consistent with AoS (12). In aphasic patients with severe AoS, a previous study found that the left lip region of M1 could be a more important target for A-tDCS to improve articulatory ability (13). How brain network changes after tDCS in post-stroke patients with severe AoS was still unknown. In this study, we focus on network connectivity changes following M1-tDCS treatment.

Modern theoretical perspectives propose that speech perception and production might be more accurately characterized by a large network of interacting brain areas rather than local independent modules (14). In this network, many brain regions contribute to speech processing. The dorsal language pathway is a classic model in speech perception and speech production, which has evidence from fMRI (15) and tractography (16). It plays a critical role in repetition tasks, transforming sensory representations into articulatory representations (17, 18). The left-dominant dorsal language pathway projects from the posterior superior temporal gyrus (STG) and includes the Sylvian parietal temporal region (SPT), inferior parietal lobe (IPL), PM, M1, inferior frontal areas (Broca's area), and their connections, *via* the arcuate and superior longitudinal fasciculi. SPT, defined as an area at the posterior Sylvian fissure around the anterior end of the temporoparietal junction (TPJ), is involved in the translation and integration between sensory codes and the motor system (19). The IPL is a region where multiple sensory inputs integrate and is involved in motor program selection (20) and motor learning (21). The left IFG (Broca's area) was strongly associated with word retrieval and speech motor programming (14, 22–25). The left PM and left M1 serve as a sequentially organized common final pathway for generating specific movement commands following projections of information from other cortical and subcortical areas (26). Not only are there language-specific networks, but there are also domain-general networks that contribute to speech processing since the language and speech system itself is an advanced cognitive function. The bilateral dorsolateral prefrontal cortex (DLPFC) is an important site of the domain-general network. It has been demonstrated that the DLPFC is activated during speech tasks and is involved in working memory, attention, and the execution of various types of information, including verbal and nonverbal information (27, 28). Each site in the networks has unique and complementary functions, and they constitute effective connectivity underlying speech production.

Several studies looked at how functional connectivity in speech networks differed in patients with AoS to uncover the neurobiological mechanisms of AoS (29, 30). For example, New and colleagues found that patients with AoS had reduced connectivity between bilateral PM as well as between the left PM and the right anterior insula (aINS) (29). However, these studies exploring functional connectivity in patients with AoS were cross-sectional studies without longitudinal comparisons. It is still not well-characterized how tDCS can alter functional connectivity in patients with AoS.

There have been several techniques developed for measuring functional network connectivity. Electroencephalogram (EEG) can record the electrical activity evoked by the cortical functional activity directly and dynamically. Non-linear dynamics analysis can characterize the neural networks underlying EEG and provide a powerful tool for studying the dynamic changes and abstracting correlations within cortical networks (31). Among possible non-linear dynamics measures, cross approximate entropy (C-ApEn) was used to analyze two related time series and measure their degree of asynchrony by comparing sequences from one series to those of the second series to reflect the spatial decorrelation of cortical potentials from two remote sites (32). Our previous study showed that the EEG non-linear index of C-ApEn revealed altered cortical interconnections after tDCS treatment in patients with prolonged disorders of consciousness (33). As the EEG examination is easy to operate and the word repetition tasks during EEG can be completed within a short time, EEG is a suitable tool for dynamically observing network activity in research and clinical practice. As a result, in this study, the EEG index of C-ApEn was used to investigate functional network connectivity.

**Figure 1:**
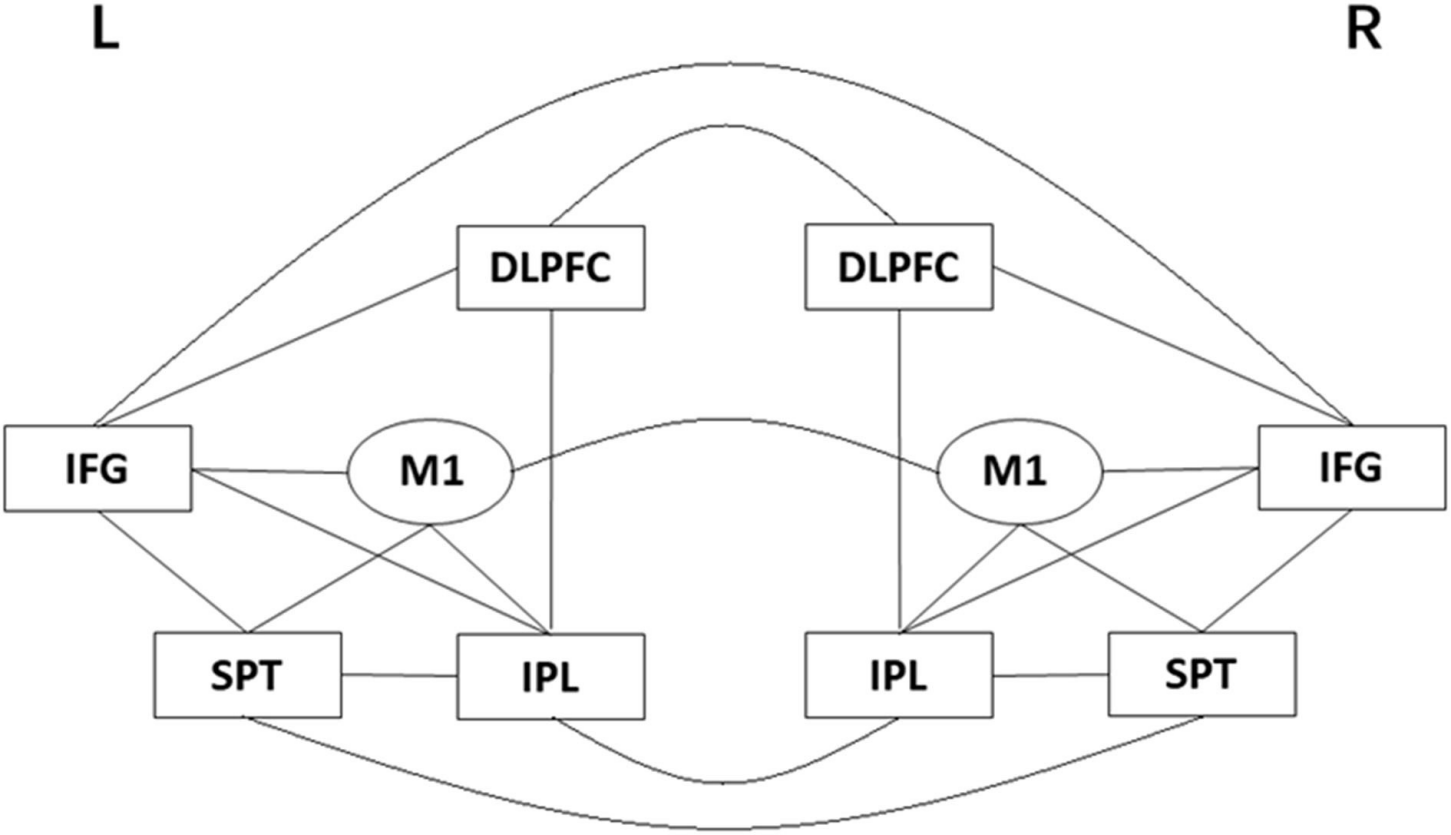
A schematic diagram of the core speech articulation network of AOS. DLPFC, dorsolateral prefrontal cortex; IFG, inferior frontal gyrus; IPL, inferior parietal lobe; M1, primary motor cortex; SPT, Sylvian-parietal-temporal region.

In a previous study, we compared the clinical effects of tDCS over M1, over Broca's area, and sham tDCS in patients with subacute post-stroke AoS (13). This study further focused on the network connectivity recorded by EEG in the M1-tDCS group and sham tDCS group. Based on the aforementioned speech articulation network, the following sites were selected as the EEG recording points: bilateral IFG, M1, IPL, SPT, and DLPFC (see Figure 1). Using non-linear EEG assessment and C-ApEn, we investigated the functional connectivity changes in the cortical speech articulation network before and after the treatment in the two groups.

## Materials and methods

### Subjects

Patients with aphasia and AoS caused by a left hemispheric stroke were recruited at the Department of Rehabilitation, Wangjing Hospital of China Academy of Chinese Medicine Sciences, and Xuanwu Hospital of Capital Medical University, Beijing, China, from January 2013 to January 2021. The inclusion criteria were as follows: (1) aged 18–80 years; (2) right-handed native Chinese speaker, assessed by the Edinburgh Handedness Inventory (34); (3) 1–4 months after stroke onset; (4) a single left hemispheric lesion involving frontal lobe; (5) no history of previous brain injury; (6) slow speech, laborious pronunciation; and (7) inability or difficulty in word repetition (monosyllabic and disyllabic word repetition score <5/10 (test scores/total scores) to avoid ceiling effect after treatment). The exclusion criteria included: (1) severely damaged auditory comprehension (auditory word-picture identification <6/30); (2) a history of seizures within 12 months until enrollment; and (3) a psychiatric disorder or dementia. The Ethics Committees of both hospitals approved this study. All participants or their guardians provided written informed consent.

### Procedure

**Figure 2:**
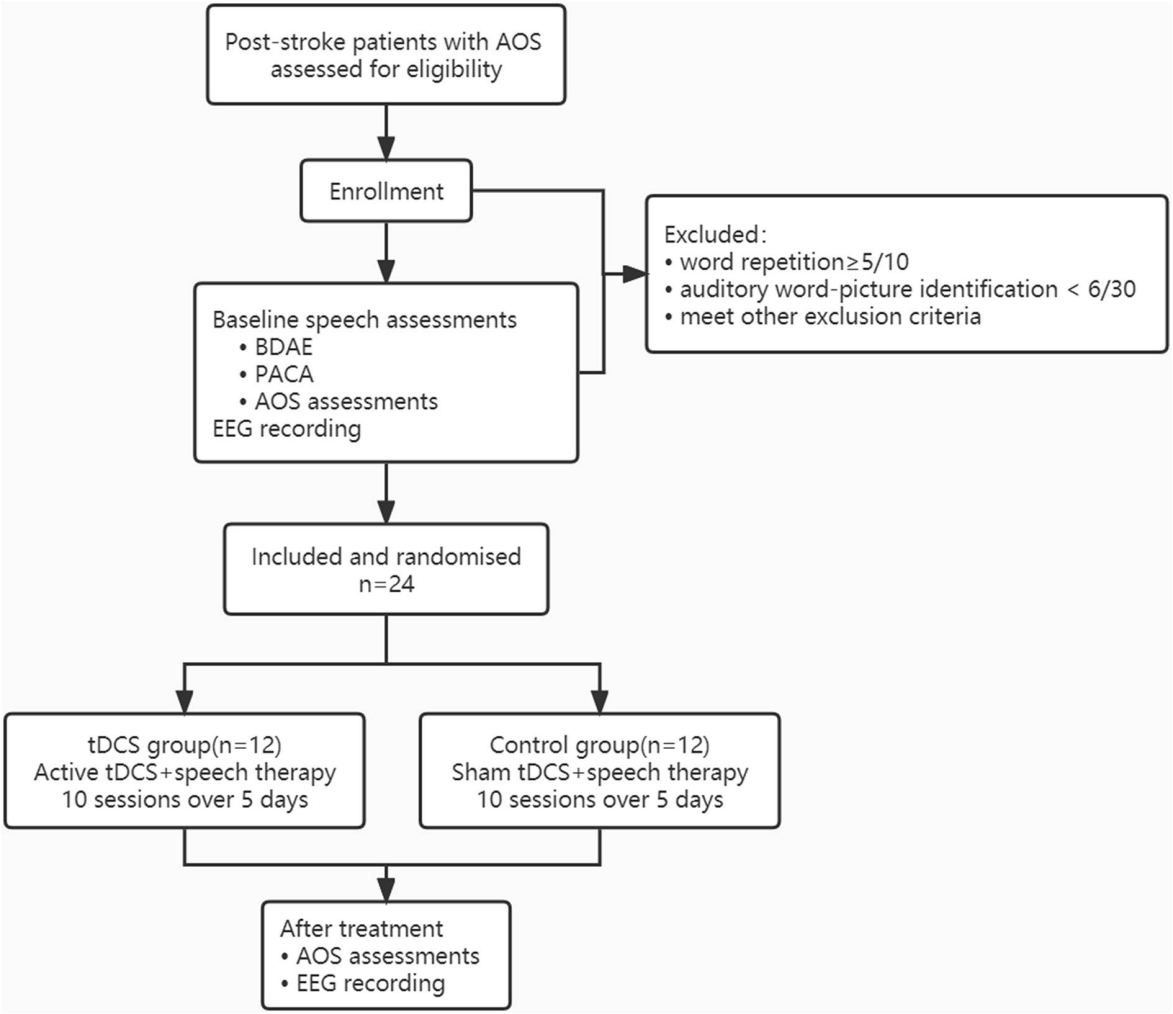
The flowchart of this study. AOS, Apraxia of speech; BDAE, Boston Diagnostic Aphasia Examination-Chinese Version; PACA, Psycholinguistic Assessment in Chinese Aphasia; tDCS, transcranial direct current stimulation; EEG, electroencephalogram.

This was a double-blind, sham-controlled, randomized controlled study. According to our previous study (13), the sample size was determined on the following parameters: α = 0.05, 1-β = 0.9, the mean difference of EEG metrics was 0.08, and the standard deviation of difference was 0.08. Then, the effect size dz was 1.0. The sample size was 11 for each group and the actual power is 0.924 (G^*^power, version 3.1.9.4). Our study included 12 cases for each group. All enrolled participants had baseline speech assessments before inclusion, including the Boston Diagnostic Aphasia Examination-Chinese Version for aphasia type and severity and Psycholinguistic Assessment in Chinese Aphasia (35) for the performance of auditory comprehension (participants with auditory word-picture identification <6/30 were excluded). Finally, a total of 24 patients were enrolled in the study and randomly assigned to one of the two groups: A-tDCS over the left lip region of the M1 (tDCS group) and sham tDCS (control group). Both groups received tDCS /sham tDCS and conventional speech and language therapy (SLT) two times per day for five consecutive days. Before and after the treatment, the AoS assessment in Chinese and the EEG was assessed. Figure 2 shows the flowchart of this study.

### Blinding

According to the computer-generated randomization sequence, the patients were divided into two groups. The assigned random number was input into the tDCS device, and then the device automatically generated an active or sham tDCS. The researchers who operated the randomization were not involved in other parts of the study. Treatment with tDCS, clinical assessments, and SLT was handled by three different language therapists with at least 10 years of experience. All participants and language therapists were blinded to the group assignment.

### tDCS

A portable battery-driven device (IS200, Chengdu, China) was used to deliver a constant current of 1.2 mA (approximately 0.05 mA/cm^2^) for 20 min to the left lip region of M1 by using a pair of saline-soaked surface sponge electrodes (4.5 × 5.5 cm). The location of the left lip region of M1 was identified where maximal amplitude motor evoked potentials were induced in orbicularis oris muscle through transcranial magnetic stimulation (TMS) (13). Over the right shoulder, the cathodal electrode was placed.

The device was turned off automatically after the 30s for the sham tDCS group, and the electrodes were placed in the same locations as the tDCS group. For both active and sham tDCS, the current intensity increased and decreased gradually. Thus, neither researchers nor patients could tell whether the stimulation they received was real or sham.

### Speech and language assessments and SLT

The type and severity of aphasia were assessed at baseline by Boston Diagnostic Aphasia Examination-Chinese Version. The patients' auditory comprehension abilities were assessed at baseline using auditory word-picture identification in Psycholinguistic Assessment in Chinese Aphasia (35). Based on Apraxia Battery for Adults (Second Edition) (36), we developed the AoS assessments in Chinese, which included counting numbers from 1 to 10 (0–10 scores); the imitation of face, tongue, and lip movements (0–20 scores); repetition of 20 Chinese phonetic alphabets (0–20 scores); repetition of 10 monosyllabic words (0–10 scores); and 10 disyllabic words (0–10 scores). Our previous article described the detailed operation (13).

SLT was applied two times per day, 30 min a time, for 5 consecutive days. The articulatory movements began with simple, visible, and fewer motor units, and the difficulty and length of the articulation were gradually increased. The training items began with simple vowels, then shifted to consonants; and afterward, the consonant was combined with a simple or compound vowel to form one syllable (a character in Chinese). Then, two-syllable words were tried for repetition. A picture was presented by the computer, the sound of the simple word was listened to and repeated two to three times, and the patients were asked to watch the speech-language pathologist's (SLP) oral movement, and repeat the word with the SLP. Gradually, auditory cues, visual cues, and speech movement control were decreased, and the training was transferred to picture naming in a stepwise manner. The training materials were different from assessment tasks, so there would not be learning effects in assessments. Our previous article described the detailed operation (13).

### EEG recording

A wireless digital EEG system (ZN16E, Chengdu, China) was utilized to complete the EEG recording (bandwidth, 0.3–100 Hz; sample rate, 500 Hz). In this study, 16 EEG electrodes were placed according to the international 10–20 system, with the exception that F7 was placed on the left IFG, which was defined as the crossing point between T3-Fz and F7-Cz, (37), and F8 was placed on the right IFG. The cortical regions corresponding to the electrode's location were as follows: DLPFC at F3/F4, IFG at F7/F8, M1 at C3/C4, SPT at T5/T6, IPL at P3/P4 (38, 39). Reference electrodes were placed on earlobes. The operation process of EEG recording and the methods to avoid electromyography artifacts and exclude the electrical noise were the same as described in previous studies (33, 40). The EEG was recorded under two conditions: eyes closed for about 5 min, followed by the eyes-closed repetition of a three-syllable word list. Due to severe AoS, performing three-syllable word repetition was challenging for some patients. Therefore, these patients were allowed to speak three monosyllable repetitions (e.g., wu-wu-wu) for the same rhythm.

### Non-linear index: Cross approximate entropy (C-ApEn)

The degree of coupling between two signals was measured using cross approximate entropy (C-ApEn) (32). A higher C-ApEn value indicated more cortical interconnections (41). The expression formula and parameters had been described in our previous studies (33, 40).

In this study, these sites in EEG were chosen as the components in the speech articulation network: DLPFC (F3/F4), IFG (F7/F8), M1 (C3/C4), IPL (P3/P4), SPT (T5/T6). The C-ApEn of these sites, including C-ApEn within the left hemisphere, C-ApEn within the right hemisphere, and inter-hemispheric C-ApEn, were calculated to illustrate the functional connectivity in the speech articulation network (Figure 1).

### Statistical analysis

The statistical software (IBM, Armonk, NY, USA) SPSS 22.0 was used to analyze the data. Pearson's chi-square test was used to determine the difference between the two groups for categorical variables. For continuous variables, the independent *t*-test (normal distribution) or Mann–Whitney U-test (non-normal distribution) was applied to test the difference between the two groups. The paired *t*-test or the paired Wilcoxon signed-rank test was adopted for comparing baseline and post-treatment for each group. The univariate and multivariate linear regression analyses were used to investigate the relevant factors for improving word repetition (the sum of monosyllable and disyllable word repetition). The group and baseline characteristics were included as independent variables in the model. The multivariate linear regression analysis included variables with *P* < 0.2 in the univariate linear regression analysis. Two-tailed *P*-values of <0.05 were considered statistically significant.

## Results

A total of 24 patients (19 men and five women; average age: 49.8 years, range: 24–73 years) with post-stroke AoS were included in the study. There was no severe adverse effect or withdrawal from the study. All the patients were asked about their feelings during the stimulation. Three patients reported slight itching or flushing in their scalps. Both groups of patients believed that they were receiving active tDCS treatment.

### Baseline

Table 1 and Supplementary Table 1 show the demographics and stroke characteristics of the participants. No significant differences were observed between groups for age, sex, education, stroke etiology, lesion site, lesion size, and post-stroke onset. The speech assessments at baseline, including types of aphasia, aphasia severity, and assessments of AoS, were similar between groups (Table 1).

**Table 1:** Clinical characteristics and speech-language assessments at baseline.

	**tDCS group (*n* = 12)**	**Control group (*n* = 12)**	***P***
Age (years)	47.42 ± 10.87	52.17 ± 14.10	0.266
Sex (male)	10(83.33%)	9(75.0%)	0.615
Education (years)	12.25 ± 2.90	10.33 ± 3.47	0.198
**Stroke etiology**			
Thrombosis MCA	12(100.0%)	11(91.67%)	0.307
Hemorrhage MCA	0(0.0%)	1(8.33%)	-
**Lesion site**			
Frontal cortex	12(100%)	12(100%)	-
Temporal cortex	12(100%)	11(91.67%)	0.307
Parietal cortex	11(91.67%)	11(91.67%)	1.000
Insula cortex	2(16.67%)	1(8.33%)	0.537
Basal ganglia	7(58.3%)	8(66.7%)	0.572
Lesion size (cm^3^)	64.42 ± 13.92	60.00 ± 11.80	0.378
Poststroke onset (weeks)	7.5 ± 3.29	5.67 ± 2.64	0.198
Aphasia type^*^			
Global	6(50.0%)	6(50.0%)	1.000
Mixed	4(33.33%)	4(33.33%)	
Broca's	2(16.67%)	2(16.67%)	
Aphasia severity^*^			
0	5(41.67%)	6(50.0%)	0.904
1	5(41.67%)	4(33.33%)	
2	2(16.67%)	2(16.67%)	
Counting numbers (score:0–10)	3.58 ± 4.17	1.67 ± 3.14	0.198
Imitation of face, tongue, and lip movements (0–20)	4.88 ± 3.00	5.38 ± 2.87	0.590
Chinese phonetic alphabet repetition (0–20)	2.58 ± 3.45	2.00 ± 3.05	0.713
Monosyllable word repetition (0–10)	1.00 ± 2.37	0.75 ± 2.01	0.932
Disyllable word repetition (0–10)	0.75 ± 2.05	0.58 ± 1.16	0.799

* Aphasia type and aphasia severity were evaluated using the Boston Diagnostic Aphasia Examination-Chinese Version.

MCA, middle cerebral artery.

Values are mean ± SD or number (percentage).

### Speech-language performance

After treatments, all five subtests of AoS assessments (counting numbers, imitation of face, tongue, and lip movements, repeating the Chinese phonetic alphabet, and repeating monosyllable and disyllable words) were improved significantly in the tDCS group. Except for the imitation of face, tongue, and lip movements, four subtests in the control group improved significantly after the treatments (*P* < 0.05; see Table 2). The changes in speech-language performance between baseline and post-treatment were compared between groups. Generally, the tDCS group had greater improvement than the control group. Significant differences were found in four of the five subtests (*P* < 0.05), while the changes in counting numbers were similar between groups (*P* > 0.05; see Table 3).

**Table 2:** Speech-language assessments of each group at baseline and post-treatment (Post-T).

	**tDCS group**				**Control group**			
	**Baseline**	**Post-T**	***P***	**Effect size**	**Baseline**	**Post-T**	***P***	**Effect size**
Counting numbers (score:0–10)	3.58 ± 4.17	7.25 ± 3.02	**0.005**	1.008	1.67 ± 3.14	5.50 ± 3.83	**0.007**	1.099
Imitation of face, tongue, and lip movements (0–20)	4.88 ± 3.00	10.50 ± 3.85	**0.002**	1.628	5.38 ± 2.87	6.58 ± 3.35	0.107	0.385
Chinese phonetic alphabet repetition (0–20)	2.58 ± 3.45	9.08 ± 4.03	**0.002**	1.733	2.00 ± 3.05	4.75 ± 2.70	**0.018**	0.955
Monosyllable word repetition (0–10)	1.00 ± 2.37	6.00 ± 3.28	**0.002**	1.747	0.75 ± 2.01	2.50 ± 2.07	**0.015**	0.858
Disyllable word repetition (0–10)	0.75 ± 2.05	4.67 ± 3.08	**0.002**	1.498	0.58 ± 1.16	2.00 ± 2.17	**0.042**	0.816

Values are mean ± SD. The bold values indicates the significant *P* values of *p* < 0.05.

**Table 3:** Changes in speech-language performance between baseline and post-treatment of each group.

	**tDCS group**	**Control group**	***P***	**Effect size**
Counting numbers (score:0–10)	3.67 ± 2.96	3.83 ± 3.30	0.977	0.051
Imitation of face, tongue, and lip movements (0–20)	5.63 ± 2.41	1.21 ± 2.10	**<0.001**	1.955
Chinese phonetic alphabet repetition (0–20)	6.50 ± 4.48	2.75 ± 3.14	**0.033**	0.969
Monosyllable word repetition (0–10)	5.00 ± 3.10	1.75 ± 1.91	**0.007**	1.262
Disyllable word repetition (0–10)	3.92 ± 2.81	1.42 ± 1.88	**0.010**	1.046

Values are mean ± SD. The bold values indicates the significant *P* values of *p* < 0.05.

The univariate and multivariate linear regression analyses were used to investigate the relevant factors for improving word repetition (the sum of monosyllable and disyllable word repetition) (Table 4). The group and baseline characteristics were included in the model as independent variables. The multivariate regression analyses showed that the tDCS group, compared with the control group, was significantly associated with better improvement in word repetition (*P*=0.002).

**Table 4:** Linear regression analysis of the relevant factors for the word repetition (the sum of monosyllable and disyllable word repetition).

	**Univariate**						**Multivariate**				
**Characteristics (ref)**	**Unstandardized coefficient**		**Standardized coefficient**	***t***	***P***	***R^2^***	**Unstandardized coefficient**		**Standardized coefficient**	***t***	***P***
	***B***	**Standard error**	***b***				***B***	**Standard error**	***b***		
Group (control)	6.250	2.024	0.550	3.088	**0.005**	0.303	6.804	1.865	0.599	3.648	**0.002**
Age	−0.083	0.097	−0.179	−0.853	0.403	0.032					
Sex (male)	−1.884	2.956	−0.135	−0.637	0.530	0.018					
Education	0.441	0.366	0.249	1.206	0.241	0.062					
Lesion size	−0.135	0.092	−0.298	−1.466	**0.157**	0.088	−0.100	0.105	−0.220	−0.947	0.356
Poststroke onset	−0.186	0.402	−0.098	−0.462	0.649	0.010					
Aphasia type	2.425	1.541	0.318	1.574	**0.130**	0.101	−0.757	2.781	−0.099	−0.272	0.788
Aphasia severity	3.090	1.511	0.400	2.044	**0.053**	0.160	2.873	2.756	0.371	1.042	0.310

The effect size for multivariate regression analysis: R^2^ 0.503, adjust R^2^ 0.399. The bold values indicates the significant *P* values of *p* < 0.05.

### Non-linear EEG analysis

**Figure 3:**
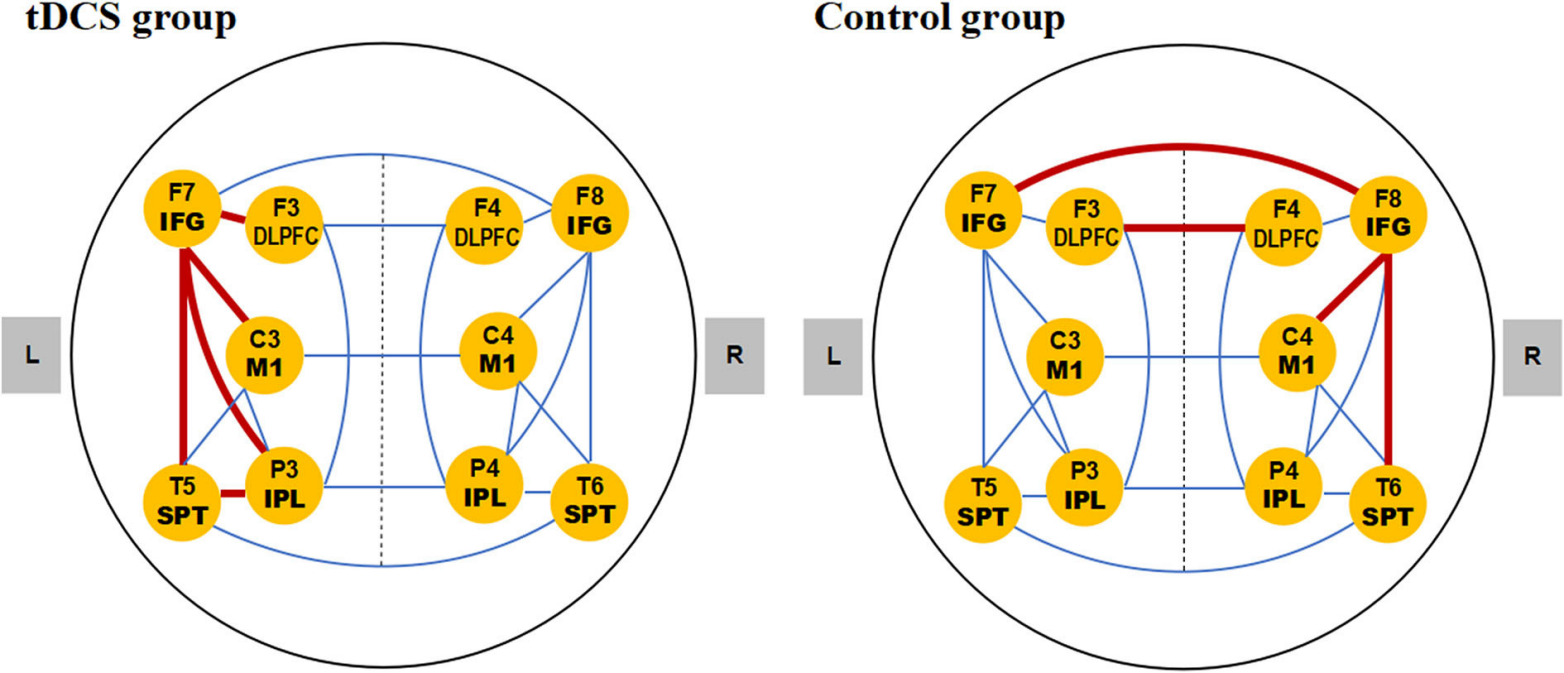
The functional connectivity significantly activated in two groups. The lines indicated the C-ApEn within the left hemisphere, within the right hemisphere, and between hemispheres, illustrating the functional connectivity in the speech articulation network. The connectivity significantly activated in two groups (results in Table 5) was marked by red bold lines. DLPFC, dorsolateral prefrontal cortex; IFG, inferior frontal gyrus; IPL, inferior parietal lobe; M1, primary motor cortex; SPT, Sylvian-parietal-temporal region.

The difference values of the C-ApEn under the resting condition and repetition task at baseline were similar between groups (*P* > 0.05). In the tDCS group, after treatment, the difference values of C-ApEn were significantly higher than those at baseline in F3-F7 (left DLPFC-left IFG), F7-C3 (left IFG-left M1), P3-F7 (left IPL-left IFG), T5-P3 (left SPT-left IPL), and T5-F7 (left SPT-left IFG). In the control group, the differences were significantly observed in F3-F4 (bilateral DLPFC), F7-F8 (bilateral IFG), T6-F8 (right SPT-right IFG), and F8-C4 (right IFG-right M1), as shown in Table 5, Figure 3. The changes in the difference value of C-ApEn were compared between the two groups (Table 6). The changes in C-ApEn in the tDCS group were overall higher than that in the control group in the left hemisphere, significantly in F3-F7 (left DLPFC-left IFG), P3-F7 (left IPL-left IFG), T5-F7 (left SPT-left IFG), T5-C3 (left SPT-left M1), and T5-P3 (left SPT-left IPL).

**Table 5:** The difference value of the cross-approximate entropy (C-ApEn) under the eye-closed condition and repetition task before and after the treatment.

	**tDCS group**				**Control group**			
	**Baseline**	**Post-T**	***P***	**Effect size** **(Cohen's *d*)**	**Baseline**	**Post-T**	***P***	**Effect size** **(Cohen's *d*)**
F3-F7	0.03 ± 0.05	0.12 ± 0.09	**0.015**	1.236	0.02 ± 0.03	0.01 ± 0.06	0.813	0.211
F3-P3	0.05 ± 0.04	0.10 ± 0.07	0.099	0.877	0.03 ± 0.03	0.05 ± 0.05	0.237	0.485
F7-C3	0.03 ± 0.05	0.11 ± 0.09	**0.013**	1.099	0.02 ± 0.03	0.03 ± 0.06	0.532	0.211
P3-F7	0.05 ± 0.04	0.12 ± 0.07	**0.010**	1.228	0.03 ± 0.04	0.03 ± 0.07	0.555	0.000
P3-C3	0.05 ± 0.04	0.10 ± 0.07	0.099	0.877	0.04 ± 0.03	0.05 ± 0.05	0.281	0.243
T5-C3	0.05 ± 0.04	0.10 ± 0.08	0.075	0.791	0.05 ± 0.04	0.04 ± 0.04	0.953	0.250
T5-P3	0.05 ± 0.04	0.11 ± 0.08	**0.050**	0.949	0.05 ± 0.05	0.04 ± 0.05	0.341	0.200
T5-F7	0.05 ± 0.04	0.12 ± 0.08	**0.012**	1.107	0.03 ± 0.03	0.03 ± 0.05	0.726	0.000
F4-F8	0.02 ± 0.09	0.05 ± 0.10	0.844	0.315	−0.01 ± 0.06	0.02 ± 0.06	0.109	0.500
F4-P4	0.05 ± 0.06	0.06 ± 0.06	0.646	0.167	0.00 ± 0.04	0.03 ± 0.06	0.074	0.588
F8-C4	0.02 ± 0.09	0.06 ± 0.07	0.181	0.496	−0.01 ± 0.05	0.04 ± 0.06	**0.016**	0.905
P4-F8	0.03 ± 0.07	0.06 ± 0.07	0.306	0.429	0.00 ± 0.05	0.03 ± 0.06	0.054	0.543
P4-C4	0.05 ± 0.08	0.07 ± 0.06	0.504	0.283	0.01 ± 0.04	0.05 ± 0.06	0.068	0.784
T6-C4	0.03 ± 0.08	0.07 ± 0.08	0.396	0.500	0.00 ± 0.04	0.03 ± 0.06	0.114	0.588
T6-P4	0.04 ± 0.09	0.08 ± 0.08	0.346	0.470	0 ± 0.06	0.03 ± 0.06	0.154	0.500
T6-F8	0.02 ± 0.09	0.05 ± 0.09	0.432	0.333	−0.02 ± 0.05	0.03 ± 0.05	**0.018**	1.000
F3-F4	0.03 ± 0.04	0.07 ± 0.06	0.055	0.784	0.00 ± 0.04	0.03 ± 0.05	**0.045**	0.663
F7-F8	0.02 ± 0.06	0.06 ± 0.08	0.229	0.566	−0.02 ± 0.07	0.02 ± 0.05	**0.044**	0.658
C3-C4	0.03 ± 0.05	0.07 ± 0.05	0.116	0.800	0.02 ± 0.03	0.04 ± 0.05	0.155	0.485
P3-P4	0.06 ± 0.04	0.08 ± 0.05	0.266	0.442	0.02 ± 0.03	0.04 ± 0.05	0.195	0.485
T5-T6	0.05 ± 0.05	0.08 ± 0.05	0.109	0.600	0.02 ± 0.04	0.04 ± 0.05	0.331	0.442

Values are mean ± SD. The bold values indicates the significant *P* values of *p* < 0.05.

**Table 6:** The comparison of changes in the difference value of the cross-approximate entropy (C-ApEn) between two groups.

	**tDCS group**	**Control group**	***P***	**Effect size**
				**(Cohen's *d*)**
F3-F7	0.09 ± 0.10	0.00 ± 0.06	**0.033**	1.091
F3-P3	0.05 ± 0.09	0.02 ± 0.04	0.478	0.431
F7-C3	0.08 ± 0.09	0.01 ± 0.07	0.060	0.868
P3-F7	0.07 ± 0.08	0.00 ± 0.06	**0.028**	0.990
C3-P3	0.05 ± 0.08	0.02 ± 0.04	0.347	0.474
T5-C3	0.05 ± 0.09	0.00 ± 0.04	**0.034**	0.718
T5-P3	0.06 ± 0.08	−0.01 ± 0.04	**0.006**	1.107
T5-F7	0.07 ± 0.08	0.00 ± 0.05	**0.024**	1.049
F4-F8	0.02 ± 0.12	0.03 ± 0.06	0.378	0.105
F4-P4	0.01 ± 0.08	0.03 ± 0.05	0.410	0.300
F8-C4	0.04 ± 0.09	0.04 ± 0.05	0.543	0.000
P4-F8	0.03 ± 0.09	0.03 ± 0.05	0.755	0.000
P4-C4	0.02 ± 0.10	0.04 ± 0.07	0.478	0.232
T6-C4	0.04 ± 0.10	0.03 ± 0.06	0.861	0.121
T6-P4	0.04 ± 0.11	0.03 ± 0.08	1.000	0.104
T6-F8	0.03 ± 0.11	0.05 ± 0.06	0.505	0.226
F3-F4	0.04 ± 0.06	0.03 ± 0.04	0.799	0.196
F7-F8	0.04 ± 0.10	0.04 ± 0.07	0.817	0.000
C3-C4	0.04 ± 0.06	0.02 ± 0.05	0.630	0.362
P3-P4	0.03 ± 0.07	0.02 ± 0.06	0.487	0.153
T5-T6	0.03 ± 0.06	0.02 ± 0.05	0.684	0.181

The bold values indicates the significant *P* values of *p* < 0.05.

## Discussion

To our best knowledge, this study was the first randomized controlled study that investigated the alteration of functional connectivity after tDCS over the left lip region of M1 combined with SLT in post-stroke AoS and the effect of tDCS on speech function. Although both groups had achieved statistically significant improvements in AoS assessments when compared to baseline, the results showed that the tDCS group had significantly greater improvement in four of the five subtests than the control group (score changes in counting numbers were not significant between groups), strongly indicating the much more benefits of tDCS combined with SLT than SLT only. Counting numbers is an automatic oral test that is relatively easier and might be improved early in most patients with AoS, so the difference in this subtest for the two groups might not be obvious. The multivariate regression analyses revealed that the main factor associated with the improvement of word repetition was the group.

Using non-linear EEG assessment and C-ApEn, we investigated the functional connectivity changes in the cortical speech articulation network before and after the treatment in two groups. It was shown that in the tDCS group, the enhanced network connectivity was all in the left hemisphere (SPT-IFG, IFG-M1, SPT-IPL, IPL-IFG, DLPFC-IFG), which seemed to be associated with the better improvement of AoS in the tDCS group (Figure 3). In the control group, the enhanced network connectivity was mainly among homologous speech-related regions on the right hemisphere (SPT-IFG, IFG-M1) and the inter-hemispheric connectivity between bilateral DLPFC and between bilateral IFG (Figure 3). The comparison of changes in the C-ApEn between the two groups (Table 6) showed that the activation of functional connectivity in the tDCS group was overall higher than that in the control group in the left hemisphere. The connectivity with *P* < 0.05 was mainly the same as the results of the comparison between pre and post-treatment in the tDCS group.

### Alteration of network connectivity in the tDCS group after treatment

In the tDCS group, the enhanced network connectivity of left SPT-left IFG and left IFG-left M1 might suggest that left IFG (Broca's area) mediates the transformation of auditory codes in the temporal cortex to articulatory movement in the motor cortex (42). The connectivity of left SPT-left IFG could be interpreted as an explanation that the function of Broca's area, which is related to speech motor programming, might also need support from temporal auditory regions to confirm the spoken sounds (43). The ventral sensorimotor cortex (i.e., M1 in our study) has been recognized as a core area in the speech network, determining the extent of network interactions (44). The activation of left IPL-left IFG might represent the interaction of two regions in word repetition tasks. IPL and the somatosensory cortex, involved in multi-sensory integration and auditory-motor feedback, are essential in adjusting parameters during a speech, coordinating speech production, and monitoring to prevent speech errors (24, 45). The connectivity of left SPT-left IPL might reflect the projection from the temporal regions where auditory representations are stored to the multi-sensory regions in the parietal lobe.

The study results showed that DLPFC was involved in the significantly activated connectivity in both groups, which means that DLPFC-related networks might be activated during the AoS recovery process, whether under the intervention of SLT alone or SLT plus tDCS. Differing from other nodes in this study, DLPFC is not a brain area specific for speech and language but serves as a crucial site in the domain-general network, involved in selective attention and cognitive control (28, 46). The loss of connection to DLPFC leads to the reduction of error monitoring (47). The activation of left DLPFC-left IFG in the tDCS group might indicate that after tDCS treatment, left DLPFC was involved in selection among competing words and error monitoring, showing that the network connectivity of patients in the tDCS group tended to be normalized.

The effects of tDCS were not limited to the stimulating electrode's location but also in a network of brain regions that are function-related. Our previous research (13) showed the increasing cortical excitability in both stimulated (M1) and non-stimulated sites (DLPFC and IFG) after M1-tDCS. Furthermore, this study showed that the stimulation over M1 promoted the connectivity of the anterior and posterior speech articulation network in the left hemisphere (IFG-SPT-IPL). However, M1 was found only connected with left IFG, while the connectivity of M1 with posterior speech-related networks (SPT, IPL) was not recovered. The reasons might be: (a) most patients have both cortical and subcortical damages, resulting in damage to the white matter connectivity between M1 and other brain regions and then the loss of functional connectivity. Therefore, it is difficult to recover the functional connectivity between M1 and most brain regions through short-term tDCS stimulation. However, this explanation needs to be confirmed by further research; (b) the small sample size of the study was another explanation and there might be bias caused by individual differences. Future research is needed to further reveal the tDCS after-effects on speech-related cortical excitability, speech-related network connectivity, and their relations with changes in speech behavior, specifically the excitability in and connectivity among the SPT, IPL, and M1.

### Alteration of network connectivity in the control group after treatment

In the control group, the network connectivity was mainly among the regions in the right hemisphere (right SPT-right IFG, right IFG-right M1), between bilateral DLPFC and bilateral IFG (see Figure 3). The connectivity of right SPT-right IFG and right IFG-right M1 in the control group was mirrored in the connectivity of left SPT-left IFG and left IFG-left M1 in the tDCS group, which might reflect the temporary compensation for the damaged speech network in the left hemisphere. Whether the subsequent recovery might shift to the left hemisphere dominant still needs to be confirmed.

There is a complex relationship between bilateral IFG. Right IFG was found suppressed during normal speech production and activated following the damage of left IFG. Moreover, this greater activity in the right IFG was associated with speech recovery without intervention (48), as the compensative activation of right IFG appeared after SLT in the control group in our study. The activation of connectivity between bilateral DLPFC might be explained by that, as the speech-specific network in the left hemisphere was less activated, domain-general networks became upregulated in language processing to adapt to the increasing speech performance demand after brain damage.

### Hypothesis of speech and language recovery

This research found that better AoS improvement in the tDCS group appeared to be related to improved network connectivity in the left hemisphere. The function of the left and right hemispheres in the recovery from post-stroke aphasia has always been debated. Some researchers have suggested that it might be maladaptive with the right hemisphere involved in the recovery, such as the transcallosal disinhibition hypothesis (49, 50). However, others consider it to be positive (51, 52). Because the right hemisphere's homologous speech-related regions have a similar but weak function to the left hemisphere's regions, the right hemisphere may become activated after the left hemisphere is damaged. Several studies showed that the activation of the right hemisphere was related to the recovery of comprehension and speech production (53, 54). The literature has proposed that the engagement of quiescent regions was one of the fundamental principles of language recovery hypotheses (55). Following damage to the network of the left hemisphere, quiescent regions of the right hemisphere may become active in speech processing to support language functions. According to the model of Directions Into Velocities of Articulators (DIVAs), the right ventral premotor cortex (vPMC) plays a part in correcting motor commands, which is considered a function of feedback control (1). This study found an improvement in AoS assessments for patients in the control group, as well as recovery of network connectivity in the right hemisphere (right SPT-right IFG, right IFG-right M1) and between bilateral DLPFC and bilateral IFG, indicating that right hemisphere involvement may be positively associated with AoS recovery to some extent.

Studies supporting the view of maladaptation found that the activation of the right hemisphere was negatively related to speech performance in the chronic stage after stroke (49, 56, 57). This negative relationship is sometimes interpreted as support for a “regional hierarchy” theory of recovery (58), according to which best speech recovery is associated with left-dominant activation, while right hemisphere involvement is a suboptimal choice. A recent review suggested that compensatory activity in the non-lesioned hemisphere leads mostly to unfavorable outcomes and further aggravated inter-hemispheric imbalance. Balanced inter-hemispheric activity with increased intrahemispheric coherence in the lesioned networks correlates with improved post-stroke recovery (59). According to one longitudinal fMRI study, the right hemisphere's contribution to recovery from stroke-induced aphasia was relatively small, and the recovery was more likely driven by the left frontotemporal networks that had previously engaged in speech and cognition (60). Temporary compensation observed in the right hemisphere might not be as significant as previously proposed (60), whereas, in the chronic phase, the activation returning to the left hemisphere was the main driving force behind the recovery.

The studies mentioned above were for post-stroke aphasia; however, studies of recovery mechanisms specific to AoS were scarce. Although both groups improved clinically compared to baseline, the tDCS group improved significantly more, and the improved network connectivity in the tDCS group was all in the left hemisphere, suggesting that the better recovery may be associated with the activation of functional connectivity among multiple speech-related regions in the left hemisphere. Therefore, we speculated that, although right hemisphere involvement might be positively associated with AoS recovery to a limited extent which mainly happened in the initial or subacute phases, left hemisphere-dominant activation might be optimal for better recovery. With tDCS, the right hemisphere's compensation period may be shortened, and the recovery process may move quickly into the stage of left-dominant activation.

To date, there have been few reports of the functional connectivity of networks about AoS recovery in literature. In light of ROI-based network analysis for patients with AOS, it was found that better continuous improvement for the long term was associated with the upregulation of the left hemisphere and inter-hemispheric connectivity (61). Another fMRI study with aphasic patients (not specific to AoS) found that sham tDCS resulted in only connectivity changes in the right hemisphere, whereas active tDCS resulted in increased functional connectivity in the left hemisphere (30). These results were consistent with some of our findings.

### Limitations

There are some limitations. First, the sample size was relatively small. Second, the location of the cortex regions was determined using the EEG international 10–20 system, which had a low spatial resolution. However, EEG was chosen as the tool for measurement because of its advantages of low cost and short time for examination, dynamic recording, and unlimited clinical use at the bedside. For further studies, high-resolution EEG could be used to detect network changes in more regions (such as SMA). Third, the tDCS treatment period in this study was relatively short (5 days), and a better improvement may be obtained if the treatment is extended. Fourth, we did not design clinical outcomes which assess the function of DLPFC, such as error monitoring. The activation of DLPFC lacks the support of behavioral data.

## Conclusion

The use of tDCS over the left lip region of M1 in conjunction with speech therapy has been shown to increase network connectivity in both speech-specific (SPT-IFG-M1, SPT-IPL, and IPL-IFG) and domain-general (DLPFC-IFG) networks in the left hemisphere and significantly improve articulation performance in patients with post-stroke AoS. For the control group, speech therapy mainly increased the connectivity among SPT-IFG-M1 regions in the right hemisphere, as well as that between bilateral IFG and bilateral DLPFC. The better recovery for the AoS might be associated with the enhanced network connectivity in the left hemisphere induced by tDCS over the left lip region of M1.

## Data availability statement

The raw data supporting the conclusions of this article will be made available by the authors, without undue reservation.

## Ethics statement

The studies involving human participants were reviewed and approved by the Ethics Committees of Wangjing Hospital of China Academy of Chinese Medicine Sciences and Xuanwu Hospital of Capital Medical University, Beijing, China. The patients/participants provided their written informed consent to participate in this study.

## Author contributions

JZ and YL have contributed equally to the data analysis and drafting of the manuscript. XZ, YY, YC, JH, GD, and BL participated in the conduction of the study including patients' enrollment, treatment, and assessments. DW and JW designed and supervised the study, and critically revised the manuscript. All authors approved the final manuscript.

## Funding

This work was supported by the National Natural Science Foundation of China (Grant numbers 81171011 and 81572220); the Scientific and technological innovation project of the China Academy of Chinese Medical Sciences (CI2021A01410); the Key Field Project of the 13th Five-Year Plan of the China Academy of Chinese Medical Science (Grant number ZZ10-015); and the Science and Technology Projects of Beijing (Grant numbers Z121107001012144 and Z171100001017111).

## Conflict of interest

The authors declare that the research was conducted in the absence of any commercial or financial relationships that could be construed as a potential conflict of interest.

## Publisher's note

All claims expressed in this article are solely those of the authors and do not necessarily represent those of their affiliated organizations, or those of the publisher, the editors and the reviewers. Any product that may be evaluated in this article, or claim that may be made by its manufacturer, is not guaranteed or endorsed by the publisher.
