# Supplementary material for: Alteration of network connectivity in stroke patients with apraxia of speech after tDCS: A randomized controlled study
Source: Front Neurol. 2022 Sep 15;13:969786. doi: 10.3389/fneur.2022.969786 (PMC9521848; doi:10.3389/fneur.2022.969786)
Supplement: Supplementary file 1 [file Table_1.DOCX]

| Group | Subject No. | Age(years) | Sex | Education(years) | Stroke etiology | Lesion site | Lesion size(cm^3^) | Poststroke onset(weeks) | Aphasia type | Aphasia severity |
| --- | --- | --- | --- | --- | --- | --- | --- | --- | --- | --- |
| A-tDCS-M1 | 1 | 41 | Male | 16 | Thrombosis MCA | Co(F,T,P)/BG | 60.6 | 4 | Global | 1 |
|  | 2 | 58 | Male | 15 | Thrombosis MCA | Co(F,T,P)/BG | 75.3 | 12 | Global | 0 |
|  | 3 | 45 | Male | 12 | Thrombosis MCA | Co(F,T,P)/BG | 66.1 | 8 | Global | 1 |
|  | 4 | 61 | Male | 12 | Thrombosis MCA | Co(F,T,P)/BG | 76.7 | 8 | Global | 0 |
|  | 5 | 24 | Female | 9 | Thrombosis MCA | Co(F,T,P) | 47.8 | 5 | Mixed | 1 |
|  | 6 | 57 | Male | 9 | Thrombosis MCA | Co(F,T,P,I)/BG | 41.6 | 9 | Broca's | 2 |
|  | 7 | 35 | Male | 12 | Thrombosis MCA | Co(F,T,P) | 62.6 | 4 | Mixed | 1 |
|  | 8 | 55 | Male | 9 | Thrombosis MCA | Co(F,T,P)/BG | 63.3 | 8 | Mixed | 1 |
|  | 9 | 48 | Male | 9 | Thrombosis MCA | Co(F,T,P)/BG | 80.6 | 12 | Global | 0 |
|  | 10 | 56 | Male | 12 | Thrombosis MCA | Co(F,T,P)/BG | 88.6 | 12 | Global | 0 |
|  | 11 | 42 | Male | 16 | Thrombosis MCA | Co(F,T,P)/BG | 57.8 | 4 | Mixed | 0 |
|  | 12 | 47 | Male | 16 | Thrombosis MCA | Co(F,T)/BG | 52.1 | 4 | Mixed | 1 |
| S-tDCS | 1 | 61 | Male | 12 | Thrombosis MCA | Co(F,T,P)/BG | 77.4 | 6 | Global | 0 |
|  | 2 | 31 | Female | 16 | Thrombosis MCA | Co(F,T,P,I)/BG | 49.8 | 4 | Broca's | 2 |
|  | 3 | 49 | Male | 9 | Thrombosis MCA | Co(F,P,I) | 48.8 | 4 | Broca's | 2 |
|  | 4 | 52 | Male | 9 | Thrombosis MCA | Co(F,T,P) | 66.1 | 4 | Global | 0 |
|  | 5 | 56 | Male | 9 | Thrombosis MCA | Co(F,T,P) | 59.6 | 12 | Global | 0 |
|  | 6 | 24 | Female | 9 | Thrombosis MCA | Co(F,T,P) | 41 | 9 | Mixed | 1 |
|  | 7 | 54 | Male | 12 | Hemorrhage MCA | Co(F,T,P) | 55 | 4 | Global | 0 |
|  | 8 | 60 | Male | 16 | Thrombosis MCA | Co(F,T,P) | 62.2 | 4 | Global | 0 |
|  | 9 | 61 | Male | 7 | Thrombosis MCA | Co(F,T,P) | 83.4 | 5 | Global | 0 |
|  | 10 | 64 | Female | 12 | Thrombosis MCA | Co(F,T,P)/BG | 55.8 | 4 | Broca's | 2 |
|  | 11 | 41 | Male | 4 | Thrombosis MCA | Co(F,T,P)/BG | 58.8 | 8 | Mixed | 1 |
|  | 12 | 73 | Female | 9 | Thrombosis MCA | Co(F,T)/BG | 62.1 | 4 | Mixed | 1 |

Supplementary Table 1. Clinical characteristics of the two groups.

A-tDCS-M1 = anodal transcranial direct current stimulation over left lip region of the primary motor cortex; MCA = middle cerebral artery; Co = cortical; F = frontal; T = temporal; P = parietal; BG = basal ganglia; I = insula; O = occipital; S-tDCS = sham transcranial direct current stimulation
